# Potential antidiabetic effect of ethanolic and aqueous-ethanolic extracts of *Ricinus communis* leaves on streptozotocin-induced diabetes in rats

**DOI:** 10.7717/peerj.6441

**Published:** 2019-02-18

**Authors:** Mohamed A.M. Gad-Elkareem, Elkhatim H. Abdelgadir, Ossama M. Badawy, Adel Kadri

**Affiliations:** 1 Department of Chemistry, Faculty of Science and Arts in Baljurashi, Albaha University, Albaha, Saudi Arabia; 2 Chemistry Department, Faculty of Science, Al-Azhar University, Assiut, Egypt; 3 Forensic Chemistry Department, College of Forensic Sciences, Naif Arab University for Security Sciences, Riyadh, Saudi Arabia; 4 Department of Biology, Faculty of Science and Arts in Baljurashi, Albaha University, Albaha, Saudi Arabia; 5 Chemistry Department, Faculty of Science, University of Sfax, Tunisia

**Keywords:** *Ricinus communis*, Blood glucose level, Antidiabetic activity, Streptozotocin, Electrolytes, Liver enzymes

## Abstract

Recently, herbal drugs and their bioactive compounds have gained popularity in the management of diabetes mellitus (DM), which has become an epidemic disease all over the world and is especially prevalent in the Kingdom of Saudi Arabia (KSA). This study aimed to investigate the antidiabetic effect of ethanolic and aqueous-ethanolic extracts of wild *Ricinus communis* (*R. communis*) leaves in streptozotocin (STZ) induced diabetic rats. Diabetic rats were administered orally with the mentioned extracts at doses of 300 and 600 mg/kg/BW for 14 days, and the obtained results of different biochemical parameters were compared with normal control, diabetic control and standard drug glibenclamide (5 mg/kg/BW). The obtained results revealed a remarkable and significantly (*P* < 0.05) reverse effect of the body weight loss, observed when diabetic rats were treated with ethanol and aqueous-ethanol extracts at 300 mg/kg/BW. Administration of the ethanol extract at 600 mg/kg/BW significantly (*P* < 0.05) reduced the blood glucose level. A significant increase in the AST, ALT and ALP levels (*P* < 0.05) was observed in the diabetic control and in the experimental groups with glibenclamide which was also significantly (*P* < 0.05) lowered after treatment with extracts at special doses. Total proteins, albumin, total bilirubin, direct bilirubin, creatinine and urea were also investigated and compared to the corresponding controls. We showed that administration of *R. communis* extract generally significantly (*P* < 0.05) ameliorated the biochemical parameters of diabetic rats. Also, the changes in serum electrolyte profile were assessed and the results demonstrate that administration of extracts at concentration of 600 mg/kg/BW generally inhibits the alteration maintain their levels. The obtained data imply the hypoglycemic effects of this plant, which may be used as a good alternative for managing DM and therefore validating its traditional usage in KSA.

## Introduction

Diabetes mellitus (DM), a disorder of carbohydrate metabolism, is a clinical and endocrinological syndrome characterized by hyperglycemia, high glycated hemoglobin, and a high risk of morbidity and mortality (Alqahtani et al., 2013). It is caused by deficiency or diminished effectiveness of endogenous insulin or the improper use of insulin by target cells, and is characterized by deranged metabolism, hypertension, and sequelae predominantly affecting the vasculature (Agoramoorthy et al., 2008). The treatment of this pathology consists of multifactorial intervention such as regular physical activity to control heart disease and high blood pressure; glycemic control, which can be done by oral hypoglycemic agents; and therapeutic approaches to retard absorption of glucose by inhibiting the carbohydrate hydrolyzing enzymes like amylase and glucosidases (Machry et al., 2018). The World Health Organization (WHO) has reported that the Kingdom of Saudi Arabia (KSA) ranks second in the prevalence of diabetes in the Middle East region and seventh in the world (Robert et al., 2016). Also, it was mentioned that the overall prevalence of DM in KSA was highest in the central region (Riyadh) and was estimated at 23.7% (age group 30–70 years), and could reach 50% of adults by 2030 (Robert et al., 2016). There is a growing need for the development of novel strategies for DM prevention with fewer side effects, such as ethnobotanical treatments (Khavandi et al., 2013; Yuan et al., 2016). Therefore, the hypoglycemic activity of a number of plant extracts has been evaluated and confirmed in animal models (Cai et al., 2016; Liu et al., 2018).

*R. communis* L. is known as kharwaa in KSA, belongs to the family Euphorbiaceae, and is among the numerous medicinal plants used in many countries, especially in tropical Africa. It is well known that the roots of this plant were used in the treatment of nervous diseases and rheumatic affections such as lumbago, pleurodynia and sciatica (Nandkarni, 1954). In the traditional Indian medicine, the different parts of this plant have been found to covers a broad spectrum of biological effects from hepatoprotective (Visen et al., 1992), laxative (Capasso et al., 1994) to inflammation and liver disorders (Kirtikar & Basu, 1991) and diuretic (Abraham et al., 1986). Also, it was demonstrated that essential oil from the leaves of this plant possess antioxidant, antimicrobial ant cytotoxic effect (Kadri et al., 2011; Zarai et al., 2012). From the literature, there are only a few reports on the antidiabetic effects of this plant in the literature, and some of them present unsuccessful results. Recently, it was reported that root and aerial parts are mainly useful in the treatment of diabetes (Pullaiah & Naidu, 2003). Fifty percent of the ethanolic extract seeds of *R. communis* showed favorable effects not only on fasting blood glucose, but also on total lipid profile and liver and kidney functions (Shokeen et al., 2008). More recently, it was reported that oral administration of ethanolic extract of Indian *R. communis* leaves collected from the house decreased the level of cholesterol, HDL, LDL, triglyceride and insulin, while increasing SGOT, SGPT, ALP, and ACP (Mann, Singh & Gubta, 2013). To increase acceptability of this plant for human consumption against DM, we attempted to use for the first time to extract the leaves of wild *R. communis*, a mixed solvent such aqueous-ethanol in higher proportion of water (60%) with minor interfering efforts to the living cells, animals, and human beings and seem to be the most suitable solvents for the extraction because of different polarity of both solvents and therefore facilitate the extraction of chemicals that are soluble in water and/or ethanol.

Thus, this is the first study that was initiated with the aim of evaluating the effect of ethanol and aqueous-ethanol extracts of *R. communis* leaves collected from Baljurashi City, KSA, on the blood biochemical parameters and antidiabetic activity in streptozotocin-induced diabetic rats. The efficacy was compared with glibenclamide, a standard hypoglycemic drug.

## Materials & Methods

### Collection of plant materials

The leaves of *R. communis* were harvested in November 2017 from a mountain in Buljurashi *City*, Al Baha, Saudi Arabia, with coordinates 19°51′34″N 41°33′26″E. Voucher specimens with the corresponding number BRC100 were deposited at the Chemistry Department, College of Science and Arts in Baljurashi, Al Baha University. This work was supported by Deanship of Scientific Research, Project number: 71/1438, Albaha University, Kingdom of Saudi Arabia.

### Preparation of plant extract

The leaves of *R. communis* were dried at room temperature, ground into a fine powder and stored at 5 °C until needed. A total of 200 g of *R. communis* powder were added to 500 mL ethanol (96%) and a mixture of aqueous-ethanol (with ratio 60:40). When obtaining the plant extract, we followed the same method as done by Bakari et al. (2015), and then the plant extract was reconstituted with distilled water for oral administration.

### Experimental animals

Forty two male Wistar rats 12-week-old (150–160 g) were obtained from the Animal Care Center, College of Pharmacy, King Saud University, Riyadh, Saudi Arabia. Animals were maintained on a 12 h light/dark in cycle polypropylene cages (six rats in each) at the ambient temperature of 2 3  ± 2 °C and relative humidity of 50–60% with food and water provided ad libitum. All experiments were carried out according to the recommendation of Experimental Animals Ethics Committee of The King Saud University in accordance with the international standards for the handling of experimental animals. The rats were acclimatized for 1 week before the start of the experiment.

### Toxicity profile

In this study, acute toxicity study was carried according to Organization for Economic Cooperation and Development, guideline 423. A limit dose of 2,000 mg/ kg body weight/oral was used. The signs of toxic effects and/or mortality were observed 3 h after administration then for the next 48 h. The body weight was recorded for consecutive 14 days. Since the extracts were found safe up to the dose level of 2,000 mg/kg body weight, a dose of 300 and 600 mg/kg body weight of the two extracts was selected for screening of the antidiabetic activity.

### Induction of diabetes

Rats were fasted overnight and experimental diabetes was induced by intraperitoneal injection of 55 mg/kg body weight of streptozotocin (STZ; Sigma, St Louis, MO, USA) dissolved in freshly prepared citrate buffer (0.1 mol/L, pH 4.5) (Arora, Reddy & Balakumar, 2010). Fasting blood sugar for the animals was measured after 72 h using Medisafe Mini Blood Glucose Reader (TERUMO Corporation Ltd., Hatagaya, Tokyo, Japan). Rats with fasting blood sugar level more than 200 mg/dL (11.1 mmol/L) were considered as diabetic and used for the study. Rats were then allowed to develop diabetes for 14 days (Arora, Reddy & Balakumar, 2010).

### Experimental design

Oral glucose tolerance test with extracts in diabetic rats: Forty two rats (36 diabetic surviving rats and six normal rats) were divided into seven groups of six rats each. The animals were treated orally once daily for 14 consecutive days as follows.

- Group 1, normal rats were treated with distilled water and used as the negative control.

- Group 2, diabetic control rats were treated with distilled water.

- Group 3, diabetic rats were given standard drug glibenclamide (5 mg/kg body weight) (Alamin, Yagi & Yagi, 2015).

- Groups 4 and 5, served as diabetic rats given ethanolic *R. communis* extracts at doses of 300 mg/ kg and 600 mg/ kg respectively, once daily for 14 days,

- Groups 6 and 7, served as diabetic rats given aqueous-ethanol extract at doses of 300 mg/ kg and 600 mg/ kg respectively, once daily for 14 days.

Normal control rats and untreated diabetic rats received equal volumes of water in place of the extract. The body weight was measured every day and the dose was calculated accordingly.

### Collection of blood samples and estimation of biochemical parameters

Blood samples were collected from overnight fasted rats (only water allowed) after 2 weeks of treatment under diethyl ether anesthesia by cardiac puncture (good quality and large volume of blood from the experimental animals) into heparinized and non-heparinized tubes for hematological and biochemical analyses. For serum samples, blood was allowed to coagulate, followed by centrifugation at 3,000 r/min for 15 min at 4 °C to separate serum. Sera were divided into aliquots and stored at −80 °C for biochemical assay.

### Biochemical analysis

For biochemical analysis we used standard commercial kits according to the manufacturer*’s* instructions. Fasting serum glucose level was determined on day 14 by glucose oxidase-peroxidase method using the kit from RANDOX Laboratories Ltd., UK. Alanine and aspartate aminotransferase (ALT and AST) (Schmidt & Schmidt, 1963) and alkaline phosphatase (ALP) (Wright, Leathwood & Plummer, 1972) were measured using kits from Randox Laboratory Ltd., UK. Serum creatinine (Owen et al., 1954), serum sodium, potassium, chloride, phosphorus and carbon dioxide (Tietz, Prude & Sirgard-Anderson, 1994), bilirubin (Malloy & Evelyn, 1937) total protein, albumin (Spencer & Price, 1977) and urea (Marsh, Fingerhut & Miller, 1965) were determined using a commercial kit from QUIMICA Clinica Aplicada, Amposta, Spain.

### Statistical analysis

A one-way analysis of variance (ANOVA) and Tukey’s post-hoc test were performed to determine significant differences between the parameters using the SPSS 19 statistical package (SPSS Ltd. Woking, UK). Means and standard errors were calculated. Differences among the mean values of the various parameters were determined by the least significant difference test. A probability level of *P* < 0.05 was used in testing the statistical significance of all experimental data.

## Results

Both two extracts (ethanol and aqueous-ethanol) given by oral route were safe up to a dose of 2,000 mg/kg/BW, and did not show any mortality and toxic effects in the behavior of the treated animals.

Body weight of different tested groups was recorded in Table 1. There was no significant (*P* > 0.05) difference in body weight between groups before induction of diabetes. By the end of the second week after induction of diabetes, diabetes symptoms appeared including weight loss when compared with the untreated diabetic rats. A significant (*P* < 0.05) decrease (12.77%) in the body weights of diabetic rats was observed after induction of STZ into the animals. The body weight of the diabetic controls was significantly (*P* < 0.05) less in comparison to the plant-treated groups only at 300 mg/kg/BW for both extracts.

**Table 1:** Effects of *Ricinus communis* leaves extracts on body weight (g) in STZ-induced diabetic rats.

	Initial body weight (g)	Final body weight (g)
Normal rats	254.61 ± 1.60	280.11 ± 2.10^#^
Control diabetic rats (STZ)	252.50 ± 0.60	220.50 ± 1.20^*^
Glibenclamide	257.32 ± 2.80	228.82 ± 2.50^*^
STZ + Ethanol extract at 300 mg/kg/BW	257.50 ± 0.70	235.80 ± 1.71^*^
STZ + Aqueous-ethanol at 300 mg/kg/BW	255.63 ± 1.20	242.10 ± 2.72^*#^
STZ + Ethanol at 600 mg/kg/BW	256.60 ± 4.20	221.11 ± 1.80^*^
STZ + Aqueous-ethanol at 600 mg/kg/BW	258.61 ± 2.18	210.50 ± 1.21^**^

**Notes.**

±: Standard Error of the Mean. •  The data were analysed using the parametric method, ANOVA followed by Tukey’s post-hoc test. •  Significant differences each group versus normal rats are indicated; *p < 0.05, **p < 0.01. •  Significant differences each group versus Control diabetic rats (STZ) are indicated; #*p* < 0.05, ##*p* < 0.01.

**Figure 1:**
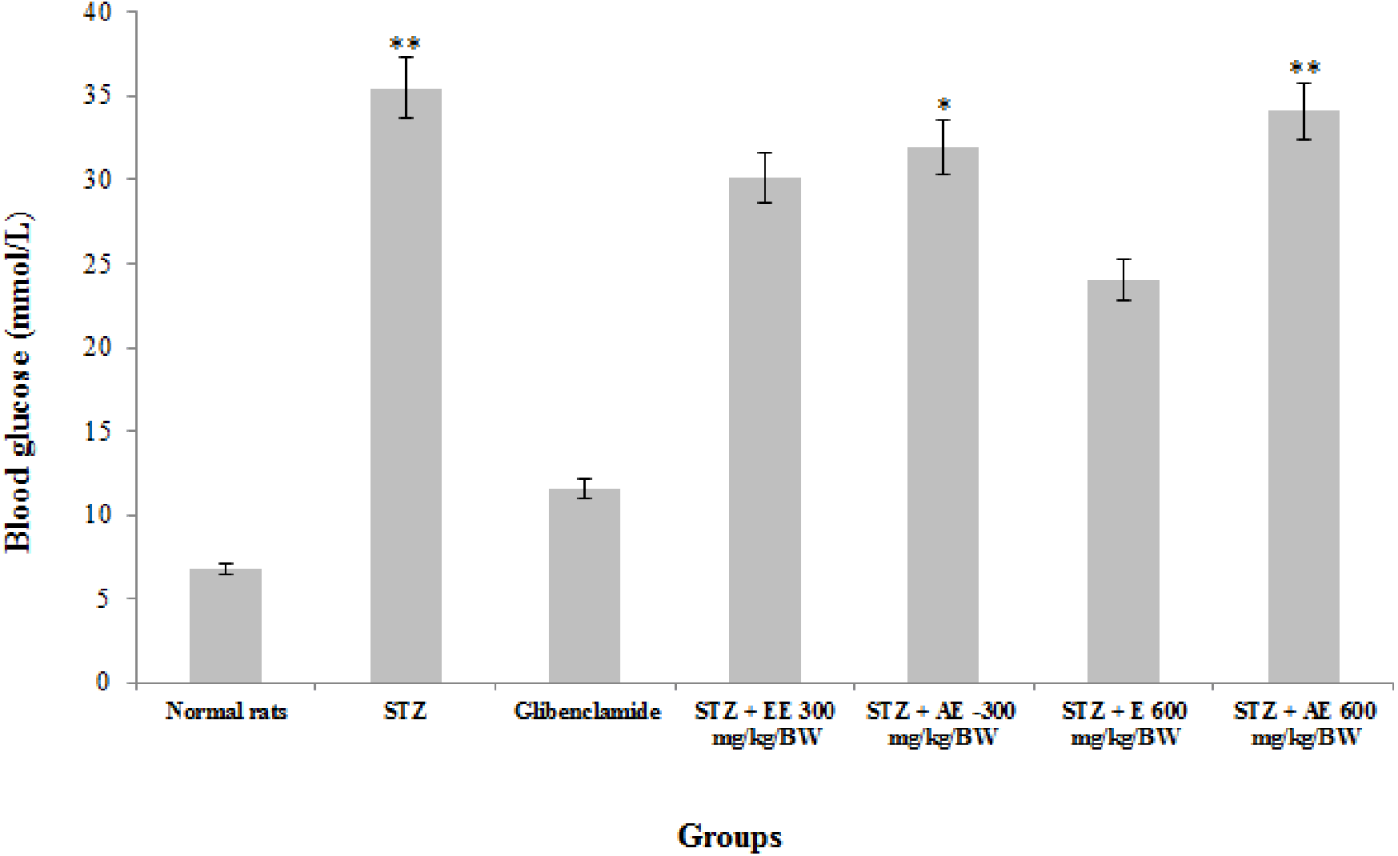
The effect of *Ricinus communis* leaves extracts on the blood glucose in STZ induced diabetic rats after 2 weeks of treatment. STZ, Control diabetic rats; STZ + EE300 mg/kg/BW, STZ + Ethanol extract at 300 mg/kg/BW; STZ + AE-300 mg/kg/BW, STZ + Aqueous-ethanol at 300 mg/kg/BW; STZ + E600 mg/kg/BW, STZ + Ethanol at 600 mg/kg/BW; STZ + AE600 mg/kg/BW, STZ + Aqueous-ethanol at 600 mg/kg/BW. *Values are statistically significant at *p* < 0.05. **Values are statistically significant at *p* < 0.01. The data were analysed using the parametric method, ANOVA followed by Tukey’s post-hoc test.

The administration of ethanol and ethanol-water extracts of *R. communis* leaves in STZ-induced diabetes in albino rats at 300 mg/kg/BW significantly (*P* < 0.05) reduced the blood glucose level in the treated rats (Fig. 1) by 15% and 10%, respectively. At a dose of 600 mg/kg/BW, this level also dramatically decreased (*P* < 0.05) by 32% for the ethanol extract and slowly for the aqueous-ethanol extract by 3.9%, respectively, compared to the positive control group. Also, the two extracts showed a comparable activity with the glibenclamide treated groups, especially with ethanol at 600 mg/kg/BW (two times lower).

From the results depicted in Table 2, we showed that AST levels significantly increased (*P* < 0.05) with glibenclamide (5 mg/kg/BW) and in the diabetic control group by a factor of 91.80% and 55.21%, respectively, as compared to the control group. Significant change (*P* < 0.05) was observed in its level in the experimental group which received, respectively, 300 mg/kg/BW of aqueous-ethanol extract and 600 mg/kg/BW of ethanol extract, but it was significantly (*P* < 0.05) lower in 300 mg/kg/BW (58.06%) and 600 mg/kg/BW (18.91%) of ethanol as compared to the control diabetic group.

**Table 2:** The effect of *Ricinus communis* leaves extracts on liver test parameters in STZ induced diabetic rats after 2 weeks of treatment.

	AST (U/L)	ALT (U/L)	ALP (U/L)	Total protein (g/L)	Albumin (g/L)	Total bilirubin (µmol/L)	Direct bilirubin (µmol/L)
Normal rats	178.20 ± 25.90	81.60 ± 25.03	283.2 ± 107.70	67.5 ± 2.23	9.55 ± 0.67^*^	1.77 ± 0.34	0.33 ± 0.06
Control diabetic rats (STZ)	276.60 ± 53.87	220.20 ± 8.39^*^	343.40 ± 26.18	60.60 ± 1.71	9.50 ± 0.23^*^	3.41 ± 0.14	0.78 ± 0.08^*^
Glibenclamide	341.80 ± 55.02	219.00 ± 16.14^*^	694.60 ± 76.47^##^	64.34 ± 1.89	7.33 ± 0.70	2.56 ± 0.05	0.72 ± 0.11^*^
STZ + Ethanol extract at 300 mg/kg/BW	175.00 ± 53.59^##^	132.00 ± 21.40^#^	678.40 ± 186.34^##^	49.64 ± 10.26	6.12 ± 1.16	1.44 ± 0.35^#^	0.18 ± 0.07^###^
STZ + Aqueous-ethanol at 300 mg/kg/BW	268.00 ± 7.34^*^	179.00 ± 9.79^#^	606.60 ± 115.37^##^	61.09 ± 0.89	6.88 ± 0.57	1.19 ± 0.15^#^	0.59 ± 0.05^##^
STZ + Ethanol at 600 mg/kg/BW	257.00 ± 43.34^*^	152.60 ± 27.04^#^	704.20 ± 155.20^##^	61.27 ± 3.50	7.80 ± 0.19	1.64 ± 0.09^#^	0.44 ± 0.14^##^
STZ + Aqueous-ethanol at 600 mg/kg/BW	232.60 ± 13.47^#^	220.20 ± 8.39^*^	923.40 ± 23.23^###^	65.40 ± 1.72	10.10 ± 0.60	3.41 ± 0.14^*^	0.76 ± 0.05^*^

**Notes.**

±: Standard error of the mean. •  The data were analysed using the parametric method, ANOVA followed by Tukey’s post-hoc test. •  Significant differences each group versus normal rats are indicated; *p < 0.05. •  Significant differences each group versus control diabetic rats (STZ) are indicated; #*p* < 0.05, ##*p* < 0.01, ###*p* < 0.001.

ALT levels (Table 2) were significantly (*P* < 0.05) elevated in the diabetic control group and in the experimental group with glibenclamide (5 mg/kg/BW) by a factor of about 2.7 as compared to the control group. Oral administration of the different extract doses significantly (*P* < 0.05) decreased the ALT levels by 66.82% and 22.90% using 300 mg/kg/BW of ethanol and aqueous-ethanol extracts, respectively, and by 44.29% using 600 mg/kg/BW of ethanol extract. However it remained invariant with 600 mg/kg/BW aqueous-ethanol extract as compared to the control diabetic group.

The ALP levels (Table 2) were dramatically enhanced (*P* < 0.05) with the group receiving glibenclamide (5 mg/kg/BW) and both extracts at 300 and 600 mg/kg/BW as compared to the control group where there was no significant change (*P* > 0.05) between the diabetic control group and the control group.

The total protein (Table 2) of the extract-treated diabetic rats was significantly (*P* < 0.05) reduced for the ethanol extract at 300 mg/kg but it remained unchanged for the others as compared to the control diabetic group.

The administration of glibenclamide (5 mg/kg/BW) lowered (*P* < 0.05) the albumin levels as compared to the normal and diabetic control (Table 2). This reduction was more pronounced (*P* < 0.05) with the ethanol extract, especially at a lower extract concentration (300 mg/kg/BW).

The total bilirubin and direct bilirubin (Table 2) were also reduced significantly (*P* < 0.05) for both extracts in STZ-induced diabetic rats at both doses except for the aqueous-ethanol extract at 600 mg/kg/BW, which was similar (*P* > 0.05) to the diabetic control.

From the results above (Table 3), induction of diabetes resulted in the elevation of serum urea and creatinine concentrations as compared to the control group and glibenclamide (5 mg/kg/BW). Compared to normal rats, no significant (*P* > 0.05) change was observed after administration of extracts, except with ethanol at a dose of 600 mg/kg/BW for creatinine and at a dose of 300 mg/kg/BW for urea (Table 3).

**Table 3:** The effect of *Ricinus communis* leaves extracts on kidney test parameters in STZ induced diabetic rats after 2 weeks of treatment.

	Creatinine (mg/dL)	Urea (mg/dL)
Normal rats	36.68 ± 4.06	6.82 ± 1.69
Control diabetic rats (STZ)	47.90 ± 1.04	12.04 ± 0.82
Glibenclamide	49.90 ± 2.15	11.32 ± 0.62
STZ + Ethanol extract at 300 mg/kg/BW	40.05 ± 4.07	6.50 ± 1.71^*##^
STZ + Aqueous-ethanol at 300 mg/kg/BW	46.72 ± 0.77	9.86 ± 0.51
STZ + Ethanol at 600 mg/kg/BW	35.27 ± 2.65	7.58 ± 0.64^#^
STZ + Aqueous-ethanol at 600 mg/kg/BW	47.17 ± 0.89	11.90 ± 0.55

**Notes.**

±: Standard error of the mean. •  The data were analysed using the parametric method, ANOVA followed by Tukey’s post-hoc test. •  Significant differences each group versus normal rats are indicated; *p < 0.05. •  Significant differences each group versus Control diabetic rats (STZ) are indicated; #*p* < 0.05, ##*p* < 0.01.

DM is frequently associated with electrolytes. The effect of the extracts on the serum concentrations of sodium, potassium, chloride and phosphorous ions and CO_2_ of the animals was assessed. The significant change (*P* < 0.05) for these parameters was observed after administration of ethanol at a dose of 300 mg/kg/BW (Table 4).

**Table 4:** The effect of *Ricinus communis* leaves extracts on chemical test parameters in STZ induced diabetic rats after 2 weeks of treatment.

	Na (mmol/L)	K (mmol/L)	Cl (mmol/L)	P (mmol/L)	CO_2_ (mmol/L)
Normal rats	139.80 ± 2.15	5.14 ± 0.28	98.80 ± 2.05	2.40 ± 0.06	29.80 ± 0.80
Control diabetic rats (STZ)	134.20 ± 2.03	6.51 ± 0.72	93.40 ± 1.16	2.27 ± 0.22	28.20 ± 0.80
Glibenclamide	135.40 ± 2.18	6.09 ± 0.09	93.60 ± 2.24	2.44 ± 0.07	28.80 ± 1.07
STZ + Ethanol extract at 300 mg/kg/BW	108.00 ± 17.90^*##^	4.3 ± 0.67^*#^	76.80 ± 1.77^*#^	1.78 ± 0.36^*#^	21.80 ± 4.00^*^
STZ + Aqueous-ethanol at 300 mg/kg/BW	136.40 ± 0.97	4.7 ± 0.05^#^	94.40 ± 0.97	2.06 ± 0.05	26.80 ± 0.48
STZ + Ethanol at 600 mg/kg/BW	140.00 ± 1.22	5.5 ± 0.13	99.80 ± 1.35	2.00 ± 0.06	27.80 ± 0.20
STZ + Aqueous-ethanol at 600 mg/kg/BW	136.40 ± 1.63	6.51 ± 0.29	93.60 ± 1.02	2.27 ± 0.22	30.60 ± 0.81

**Notes.**

±: Standard error of the mean. •  The data were analysed using the parametric method, ANOVA followed by Tukey’s post-hoc test. •  Significant differences each group versus normal rats are indicated; *p < 0.05. •  Significant differences each group versus Control diabetic rats (STZ) are indicated; #*p* < 0.05, ##*p* < 0.01.

## Discussion

N-methylnitrocarbamoyl-D-glucosamine, popularly known as streptozotocin (STZ), is a nitrosourea compound used as a screening model to evaluate the antidiabetic potential to prompt diabetes in rats. It was widely used in studies investigating of DM, as it specifically targets β-cells and reduces blood insulin levels, leading to hyperglycemia and mimicking DM pathology (Lenzen, 2008). STZ partly destroys the beta cells that secrete *inadequate* amounts of *insulin*, creating type 2 diabetes (Dimo et al., 2007). Type 2 DM induced by high-energy *diet intake* and low dose of STZ in experimental animals is considered a good model for preliminary screening of diabetic effects. Also, it is well-known that liver dysfunction is an important cause of death in patients with type 2 diabetes which plays a crucial role in maintaining blood glucose homeostasis.

Body weight is an indicator of good health and efficient metabolic homeostasis. From the results of this work, the differences in body weight between groups before induction of diabetes were not significant. The decreased of body weight in diabetic rats is due to the deficiency of insulin, the fat and protein are catabolized. The improved body weight by the extracts may be due to the alternative fats and tissue proteins that are broken down to produce energy that therefore compensate the loss in body weight and control of the hyperglycemic state in these rats and their energy intake (Atangwho et al., 2012).

Glucose is an important fuel for all cells and organs, but at high concentrations, it can cause many problems. Consequentially, blood glucose may be controlled in order to thwart the diverse damages produced by elevated glucose levels. The oral administration of ethanol and aqueous-ethanol extracts of *R. communis* leaves decrease the blood glucose level in STZ-induced diabetic albino rats. This hypoglycemic effect was more pronounced for the aqueous-ethanol extract at a dose of 600 mg/kg/BW and correlated with the study reported by Mann, Singh & Gubta (2013) for the ethanol extract. This finding suggests that both extracts exhibited a potent antihyperglycemic activity in diabetic rats and may be due to the richness of the used extract in hypoglycemic alkaloids, flavonoids and saponins which could act synergistically and/or independently as a stimulant for the release of insulin following the repair of pancreatic β-cells by the extract or by inhibition of the intestinal absorption glucose (Kibiti & Afolayan, 2015; Akinyemi et al., 2016). The glibenclamide effects on blood glucose levels act by increasing the activity of pancreatic β-cells of the pancreas result in the secretion of a large amount of insulin (Shalev, 1999).

The liver and kidney are the crucial organs in the body involved in almost all biochemical pathways such as regulating homeostasis. As shown, the enhanced levels of AST, ALT and ALP in the diabetic control and experimental groups compared to the untreated diabetic animals were related to hepatic dysfunction, such as cell necrosis of many tissues, and may be due the leakage of these enzymes and loss of functional integrity of cell membrane in liver (Swamy et al., 2018).

The significant decrease in the level of the liver parameters, especially with the ethanol extract at 300 mg/kg/BW dose, may be an indication that this concentration is the best and safest dose to use when administering this extract, and that ethanol *R. communis* extract may be used to exert a protective effect to thwart the liver damage caused by diabetes.

Serum total protein is an indication of the amount of albumin and globulin. The reduction of total protein with the ethanol extract at 300 mg/kg/BW may be a consequence of protein degradation in the tissue and the conversion of glycogenic amino acid to CO_2_ and H_2_O.

Albumin, which accounts for about 50% of* total serum protein concentration,* is a major carrier protein that circulates in the bloodstream. Therefore, a lack of serum albumin suggests chronic damage to the liver as a result of infection (Mahmoodzadah, Mazani & Rezagholizadeh, 2017). The observed decrease in albumin level for the ethanol extract and aqueous-ethanol extract at 300 mg/kg/BW may be due to the efficiency of the extract at this dose to enhance the glucose concentration which occurs due to loss of the normal feedback inhibition of gluconeogenesis in the liver followed by an increased breakdown of fats and proteins and the conversion of glucogenic amino acid to glucose (Qaid & Abdelrahman, 2016).

Bilirubin is an important metabolic product that results from the breakdown and destruction of old red blood cells. Increased serum level of bilirubin in diabetic rats in the present study was related to the uptake arising from liver disease. Administration of ethanol *R. communis* extract was able to reverse this condition in diabetic rats, thereby lowering the bilirubin level to normalcy. We explained this with the fact that extracts containing glucosides might be converted to glucorunic acid for conjugating with bilirubin for excretion (Afrisham et al., 2015; Kumar, 2017). Also, the extract can activate the Constitutive Andostane Receptor (CAR) in order to facilitate the bilirubin clearance pathway (Arthur et al., 2012).

*Creatinine* and *urea* are nitrogenous end products of metabolism which reflect glomerular filtration rate. Creatinine is produced after the pyrophosphate cleavage of phosphocreatine to produce energy for muscle activity (Stryer, Tymoczko & Berg, 1997). Urea is produced from the oxidative deamination of amino acids in which ammonia generated is transported to the liver for the formation of urea through the urea cycle. The higher level of creatinine and urea in diabetic rats when compared to the control ones is an indication of renal dysfuntion and metabolic disturbance induced by STZ diabetes (Helal et al., 2014). The significant decrease in the level of plasma urea and creatinine, especially after administration of ethanol at a dose of 300 mg/kg/BW, suggested that the extract may contain some active compounds such as polyphenols and flavonoids known for their antioxidant nephroprotective activities and their reduction of serum urea and creatinine levels.

DM was usually associated with electrolyte disorders. Electrolytes imbalance in diabetes is primarily a result of elevated blood glucose and their measurement is essential in DM to decrease their effect. From the above results, we noted the remarkable decrease of serum sodium, phosphorus, potassium, chlorine and carbon dioxide concentrations in STZ-induced diabetes in rats at 300 mg/kg/BW as compared to untreated diabetic rats. Sodium is the main extracellular electrolyte that regulates body fluid volume and electrical cell membrane potential, and is involved in the active transport of various substances in and out of cells. Low serum sodium (hyponatremia) and high serum sodium concentrations (hypernatremia) can occur in a wide variety of conditions. For sodium serum, the decreased level effect was attributed to dilutional hyponatremia (Atangwho et al., 2009). Both *R. communis* extracts suppressed generally this effect at 600 mg/kg/BW, which is an indication of a good management of DM. Chloride, along with sodium, is associated with increased risk of death in those with heart failure, hypertension or chronic disease (low chloride) or reduction in blood pH which further disturbs acid base balance due to diabetic ketoacidosis (high chloride). Changes in chloride level followed similar trend with sodium. The pronounced chloride levels at 600 mg/kg/BW might be due to diabetic ketoacidosis that induced a reduction in blood pH which further disturbs acid base balance and leads to the elevation of chloride. Phosphorus is predominantly an intracellular electrolyte and a structural component of bones and teeth. It enters cells like potassium from the extracellular fluid if the rate of glucose metabolism and utilization in the cell was enhanced. The decreased effect of phosphorus (hypophosphataemia) was probably explained by the fact that the extract at this concentration caused clearance of glucose from blood to tissue (Atangwho et al., 2009). It was reported that potassium supplementation yields improved insulin sensitivity, responsiveness and secretion (Rajendra, Narayan & Granavel, 2007). The treatment with aqueous-ethanol extract at 600 mg/kg/BW increase the phosphorus level in serum, especially with aqueous-ethanol extract which is probably due to the synergetic effect of water and ethanol to extract the suitable active molecules that contribute to this electrolyte. Potassium as the main intracellular cation was served for neurologic function, vascular tone, muscle contraction, maintenance of cellular volume, pH regulation, glycogen and protein synthesis, enzyme activity, and resting cell membrane potential.

Also, in this study we attempted to determine the carbon dioxide level which can be used to help diagnose kidney disease. The decreased in blood CO_2_ is seen in metabolic acidosis and compensated respiratory alkalosis. Treatment of STZ-induced diabetes in rats with aqueous-ethanol extract of 600 mg/kg/BW remains them to the normal state and might be useful for the management of DM.

## Conclusions

The antidiabetic activity of ethanol and aqueous-ethanol extracts of *R. communis* leaves was investigated. The obtained results imply that the ethanol extract had the highest pronounced effect in reducing the elevated blood glucose levels and in protecting the liver and kidney functions in streptozotocin-induced diabetes in rats. This study, therefore, recommends the use of ethanol *R. communis* leaf extract in the management of DM. However, further studies are required to elucidate and isolate specific active compounds responsible for the design of therapeutic alternatives for the treatment of DM.

### Limitations of the study

Some limitations of this study are the lack of analytical materials, small sample sizes used, the cross-sectional design and the time allowed to this project.

##  Supplemental Information

10.7717/peerj.6441/supp-1 Data S1 Raw Data 1

10.7717/peerj.6441/supp-2 Data S2 Raw Data 2

10.7717/peerj.6441/supp-3 Data S3 Raw Data 3

## Additional Information and Declarations

### Data Availability

The following information was supplied regarding data availability: Zenodo: https://zenodo.org/record/1828808.

## References

[ref-1] Abraham Z, Bhakuni SD, Garg HS, Goel AK, Mehrotra BN, Patnaik GK (1986). Screening of Indian plants for biological activity. Indian Journal of Experimental Biology.

[ref-2] Afrisham R, Aberomand M, Ghaffari MA, Siahpoosh A, Jamalan M (2015). Inhibitory effect of *Heracleum persicum* and *Ziziphus jujuba* on activity of alpha-amylase. Journal of Botany.

[ref-3] Agoramoorthy G, Chen F, Venkatesalu V, Kudo DH, Shea PC (2008). Evaluation of antioxidant polyphenols from selected medicinal plants of India. Asian Journal of Chemistry.

[ref-4] Akinyemi O, Iyebor EW, Osadebe CO, Oniroko NS (2016). Proximate and phytochemical compositions of *Ricinus communis* in Ibadan, South-Western Nigeria. Nutrition Research and Food Science.

[ref-5] Alamin MA, Yagi AI, Yagi SM (2015). Evaluation of antidiabetic activity of plants used in Western Sudan. Asian Pacific Journal of Tropical Biomedicine.

[ref-6] Alqahtani N, Khan WA, Alhumaidi MH, Ahmed YA (2013). Use of glycated hemoglobin in the diagnosis of diabetes mellitus and pre-diabetes and role of fasting plasma glucose, oral glucose tolerance test. International Journal of Preventive Medicine.

[ref-7] Arora MK, Reddy K, Balakumar P (2010). The low dose combination of fenofibrate and rosiglitazone halts the progression of diabetes-induced experimental nephropathy. European Journal of Pharmacology.

[ref-8] Arthur FKN, Woode E, Terlabi EO, Larbie C (2012). Bilirubin lowering potential of *Annona muricata* (Linn.) in temporary jaundiced Rats. American Journal of Pharmacology and Toxicology.

[ref-9] Atangwho IJ, Ebong PE, Egbung GE, Ani IF (2009). Effects of co-administration of extracts of *Vernonia amygdalina* and *Azadirachta Indica* on serume profile of diabetic and non-diabetic rats. Australian Journal of Basic and Applied Sciences.

[ref-10] Atangwho IJ, Ebong PE, Eyong EU, Asmawi MZ, Ahmad M (2012). Synergistic antidiabetic activity of *Vernonia amygdalina* and *Azadirachta indica*: biochemical effects and possible mechanism. Journal of Ethnopharmacology.

[ref-11] Bakari S, Ncir M, Felhi S, Hajlaoui H, Saoudi M, Gharsallah N, Kadri A (2015). Chemical composition and *in vitro* evaluation of total phenolic, flavonoid, and antioxidant properties of essential oil and solvent extract from the aerial parts of *Teucrium polium* grown in Tunisia. Food Science and Biotechnology.

[ref-12] Cai Y, Qiu R, Lu Yu, Huang C, Wang J, Ji Y, Wang A (2016). Hypoglycemic activity of two anthraquinone derivatives from *Juncus setchuensis* Buchen. International Journal of Clinical and Experimental Medicine.

[ref-13] Capasso F, Mascolo N, Izzo AA, Gaginella TS (1994). Dissociation of castor oil induced diarrhoea and intestinal mucosal injury in rat, effect of NG-nitro-Larginine methyl ester. British Journal of Pharmacology.

[ref-14] Dimo T, Rakotonirina SV, Tan PV, Azay J, Dongo E, Kamtchouing P, Cros G (2007). Effect of *Sclerocarya Birrea* (Anacardiaceae) stem bark methylene chloride/methanol extract on streptozotocin-diabetic rats. Journal of Ethnopharmacology.

[ref-15] Helal EGE, Aouf NA, Khattab AM, Zoair MA (2014). Antidiabetic effect of *Artemisia annua* (kaysoum) in alloxan-induced diabetic rats. The Egyptian Journal of Hospital Medicine.

[ref-16] Kadri A, Gharsallah N, Damak M, Gdoura R (2011). Chemical composition and *in vitro* antioxidant properties of essential oil of *Ricinus communis* L. Journal of Medicinal Plants Research.

[ref-17] Khavandi K, Amer H, Ibrahim B, Brownrigg J (2013). Strategies for preventing type 2 diabetes: an update for clinicians. Therapeutic Advances in Chronic Disease.

[ref-18] Kibiti CM, Afolayan AJ (2015). Herbal therapy: a review of emerging pharmacological tools in the management of diabetes mellitus in Africa. Pharmacognosy Magazine.

[ref-19] Kirtikar KR, Basu BA (1991). Indian med. Plants.

[ref-20] Kumar M (2017). A review on phytochemical constituents and pharmacological activities of *Ricinus communis* L. Plant. International Journal of Pharmacognosy and Phytochemical Research.

[ref-21] Lenzen S (2008). The mechanisms of alloxan- and streptozotocin-induced diabetes. Diabetologia.

[ref-22] Liu Y, Cao Y, Fang S, Wang T, Yin Z, Shang X, Yang W, Fu X (2018). Antidiabetic effect of *Cyclocarya paliurus* leaves depends on the contents of antihyperglycemic flavonoids and antihyperlipidemic triterpenoids. Molecules.

[ref-23] Machry RV, Pedroso HU, Vasconcellos LS, Nunes RR, Evaldt CA, Yunes Filho EB, Rodrigues TDC (2018). Multifactorial intervention for diabetes control among older users of insulin. Revista de Saude Publica.

[ref-24] Mahmoodzadah Y, Mazani M, Rezagholizadeh L (2017). Hepatoprotective effect of mrhanolic *tanacetum parthenium* extract on CCl4-induced liver damage in rats. Toxicology Reports.

[ref-25] Malloy HT, Evelyn KA (1937). The determination of bilirubin with the photoelectric colorimeter. The Journal of Biological Chemistry.

[ref-26] Mann S, Singh PK, Gubta AAA (2013). Antidiabetic effects of *Ricinus communis* on the blood biochemical parameters in streptozotocin induced rat. International Journal of Pharma and Biosciences.

[ref-27] Marsh WH, Fingerhut B, Miller H (1965). Automated and manual direct methods for the determination of blood urea. Clinical Chemistry.

[ref-28] Nandkarni KM (1954). Indian materia medica.

[ref-29] Owen JA, Iggo B, Scandrett FJ, Stewart CP (1954). The determination of creatinine in plasma or serum, and in urine; critical examination. The Biochemical Journal.

[ref-30] Pullaiah T, Naidu KC (2003). Antidiabetic plants in India and herbal based antidiabetic research.

[ref-31] Qaid MM, Abdelrahman MM (2016). Role of insulin and other related hormones in energy metabolism-a review. Cogent Food and Agriculture.

[ref-32] Rajendra A, Narayan V, Granavel I (2007). Study on the analysis of trace elements in Aloe vera and its biological importance. Journal of Applied Science Research.

[ref-33] Robert AA, Al Dawish MA, Braham R, Musallam MA, Al Hayek AA, Al Kahtany NH (2016). Type 2 diabetes mellitus in Saudi Arabia: major challenges and possible solutions. Current Diabetes Reviews.

[ref-34] Schmidt E, Schmidt FW (1963). Determination of serum GOT and GPT activities. Enzymologia Biologica Et Clinica.

[ref-35] Shalev A (1999). Hope for insulin mimetic oral antidiabetic drugs. European Journal Endocrinology.

[ref-36] Shokeen P, Anand P, Murali YK, Tandon V (2008). Antidiabetic activity of 50% ethanolic extract of *Ricinus communis* and its purified fractions. Food and Chemical Toxicology.

[ref-37] Spencer K, Price CP (1977). Influence of reagent quality and reaction conditions on the determination of serum albumin by the bromcresol green dye binding method. Annals of Clinical Biochemistry.

[ref-38] Stryer L, Tymoczko JL, Berg J (1997). Biochemistry.

[ref-39] Swamy SK, Nagalakshmi NC, Santhosh K, Yogesh HS (2018). Hypoglycemic activity of ethanol extract of *Jasminum grandiflorum* flowers *in vivo* and cytotoxicity of its chloroform isolate *in vitro*. Journal of Diabetes and Metabolic Disorders.

[ref-40] Tietz NW, Prude EL, Sirgard-Anderson O (1994). Tietz textbook of clinical chemistry.

[ref-41] Visen P, Shukla B, Patnaik G, Tripathi S, Kulshreshtha D, Srimal R, Dhawan B (1992). Hepatoprotective activity of *Ricinus communis* leaves. International Journal of Pharmacology.

[ref-42] Wright PJ, Leathwood PD, Plummer DT (1972). Enzymes in rat urine: alkaline phosphatase. Enzymologia.

[ref-43] Yuan H, Ma Q, Ye L, Piao G (2016). The traditional medicine and modern medicine from natural products. Molecules.

[ref-44] Zarai Z, Chobba IB, Mansour RB, Békir A, Gharsallah N, Kadri A (2012). Essential oil of the leaves of *Ricinus communis* L. *in vitro* cytotoxicity and antimicrobial properties. Lipids in Health and Disease.

